# An Emergent Nexus between Striae and Thoracic Aortic Dissection

**DOI:** 10.3390/genes13010023

**Published:** 2021-12-23

**Authors:** Benjamin J. Landis, Courtney E. Vujakovich, Lindsey R. Elmore, Saila T. Pillai, Lawrence S. Lee, Jeffrey E. Everett, Larry W. Markham, John W. Brown, Phillip J. Hess, Joel S. Corvera

**Affiliations:** 1 Riley Hospital for Children Heart Center, Department of Pediatrics, Indiana University School of Medicine, Indianapolis, IN 46202, USA; cvujakov@iupuc.edu (C.E.V.); lhelvaty@iupui.edu (L.R.E.); lwmarkha@iu.edu (L.W.M.); 2 Department of Medical and Molecular Genetics, Indiana University School of Medicine, Indianapolis, IN 46202, USA; 3 Department of Surgery, Indiana University School of Medicine, Indianapolis, IN 46202, USA; sailatpillai@gmail.com (S.T.P.); lawrlee@iu.edu (L.S.L.); jeeever@iu.edu (J.E.E.); jobrown@iupui.edu (J.W.B.); phess2@iuhealth.org (P.J.H.); jcorvera@iuhealth.org (J.S.C.); 4 Department of Medicine, Indiana University School of Medicine, Indianapolis, IN 46202, USA

**Keywords:** thoracic aortic aneurysm, thoracic aortic dissection, connective tissues, striae, Marfan syndrome

## Abstract

Current approaches to stratify the risk for disease progression in thoracic aortic aneurysm (TAA) lack precision, which hinders clinical decision making. Connective tissue phenotyping of children with TAA previously identified the association between skin striae and increased rate of aortic dilation. The objective of this study was to analyze associations between connective tissue abnormalities and clinical endpoints in adults with aortopathy. Participants with TAA or aortic dissection (TAD) and trileaflet aortic valve were enrolled from 2016 to 2019 in the setting of cardiothoracic surgical care. Data were ascertained by structured interviews with participants. The mean age among 241 cases was 61 ± 13 years. Eighty (33%) had history of TAD. While most participants lacked a formal syndromic diagnosis clinically, connective tissue abnormalities were identified in 113 (47%). This included 20% with abdominal hernia and 13% with skin striae in atypical location. In multivariate analysis, striae and hypertension were significantly associated with TAD. Striae were associated with younger age of TAD or prophylactic aortic surgery. Striae were more frequent in TAD cases than age- and sex-matched controls. Thus, systemic features of connective tissue dysfunction were prevalent in adults with aortopathy. The emerging nexus between striae and aortopathy severity creates opportunities for clinical stratification and basic research.

## 1. Introduction

Thoracic aortic aneurysm (TAA) is an aortopathy that predisposes one to life-threatening thoracic aortic dissection (TAD). From 1980 to 2014, over 500,000 deaths were attributed to aortic aneurysm in the United States [1]. Genetic etiologies of TAA include Mendelian connective tissue disorders such as Marfan syndrome (MFS) and Loeys-Dietz syndrome (LDS) as well as autosomal dominant familial TAA [2]. Early diagnosis of TAA and risk stratification provide an opportunity to intervene medically to prevent progressive dilation or surgically with prophylactic aortic repair before the onset of a TAD. Establishing a genetic diagnosis or familial predisposition may alter clinical management, including modified thresholds for earlier prophylactic aortic repair [3]. Currently, clinical genetic testing does not identify a cause for most patients with TAA. Moreover, variability in the severity of TAA, even among individuals with the same genetic diagnosis, complicates management decisions. Therefore, it is important to define additional prognostic factors for TAD in order to optimize care. Prior work characterized systemic connective tissue abnormalities in a pediatric population with TAA who did not have a genetic syndrome, finding that skin striae were associated with increased rates of aortic root dilation [4]. Neither the prevalence nor potential prognostic utility of abnormal connective tissue findings is well understood in the general adult TAA population.

In this study, we sought to characterize the non-cardiovascular connective tissue phenotypes in a population of adults with TAA who were receiving cardiothoracic surgical care. We hypothesized that connective tissue abnormalities are prevalent and are associated with increased odds of TAD.

## 2. Materials and Methods

### 2.1. Selection and Description of Participants

This was an observational study of patients with diagnosis of TAA or TAD. Participants were enrolled from January 2016 to October 2019. Inclusion criteria were: (1) TAA (diameter ≥ 3.7 cm) or prior TAD involving the aortic root or ascending aorta; (2) trileaflet aortic valve; (3) age >18 years at enrollment; and (4) no history of traumatic aortic injury or aortitis. Eligible patients were identified via clinic and operating room schedules and hospital admission lists at the tertiary care Methodist Hospital of Indiana University School of Medicine (IUSM) in Indianapolis, IN. All included patients were evaluated and managed by cardiothoracic surgery providers in conjunction with the Thoracic Aortic Surgery Program at IUSM. A healthy control group included participants who did not have TAA or TAD or a genetic syndrome and were >18 years old. All participants were enrolled as part of the larger Collaborative Human Aortopathy Repository study [5].

### 2.2. Data Collection and Outcome Measures

Structured interviews were completed by study personnel in order to obtain clinical and phenotype data directly from participants. These were accompanied by electronic medical record review. Data included demographic, lifestyle, cardiovascular, and non-cardiovascular variables. The connective tissue features that are part of the systemic criteria of the revised Ghent nosology for MFS that were ascertained for this study were pectus carinatum, pectus excavatum, spontaneous pneumothorax, scoliosis, mitral valve prolapse, and skin striae [6]. Information about the locations of striae was requested specifically in order to determine if striae were present in an atypical body location. A prior study that included dermatological examination of adults with MFS and healthy controls was utilized to define the atypical locations [7]. Striae located on the hip, thigh, or buttock were not counted as atypical. Additionally, striae on the abdomen or breast were not counted as atypical if the participant had history of pregnancy, because striae gravidarum frequently develop in these locations [8]. Interviews also ascertained connective tissue abnormalities that are less characteristic of MFS, but occur in other connective tissue disorders such as LDS and Ehlers-Danlos syndrome. These included abdominal hernia (inguinal, umbilical, or femoral), hyperflexibility, atrophic scarring, and skin hyperextensibility. All available cardiac imaging results were reviewed in order to obtain aortic diameters. Three-generation pedigrees were obtained via direct interviews. The results of clinical genetic testing were recorded. The main analysis compared the clinical characteristics, including connective tissue abnormalities, between cases with TAD versus TAA cases without TAD. The secondary analyses (1) investigated age at first aortic event (aortic surgery or TAD) or age at first TAD as secondary outcomes and (2) compared TAD cases with age- and sex-matched controls without aortopathy. Electronic data and electronic files were maintained in a Research Electronic Data Capture database at IUSM [9].

### 2.3. Statistics

Analyses were performed using the JMP 12.2.0 statistical software package (SAS Institute, Cary, NC, USA). Categorical data are reported as frequencies. Continuous variables are reported as means ± standard deviations. To compare the frequencies of categorical variables between groups, contingency tables and Pearson’s chi-squared tests were utilized. The characteristics with *p*-value < 0.05 were included for multiple logistic regression analysis. Forest plots of the log_2_ transformed odds ratios and 95% confidence intervals were created using the ‘ggplot2’ package in R software (https://www.R-project.org; accessed on 2 February 2021; current use version 4.0.4). Cumulative revised Ghent systemic criteria points [6] were modeled as an ordinal dependent variable and compared between TAD and non-TAD cases using the Cochran Armitage Trend Test. The ages at initial aortic event (TAD or prophylactic aortic surgery) were compared using the non-parametric Wilcoxon rank sum test. To compare TAD cases with controls, age- and sex-matching was performed in R (‘matching’ package) and groups were compared using McNemar’s test. Responses that were not reported or uncertain and were unable to be verified in medical records were considered as missing and excluded from analysis. The frequency of missing data was <2.5% for all variables. Statistical significance was defined as α < 0.05.

## 3. Results

### 3.1. Cohort Description

**Figure 1:**
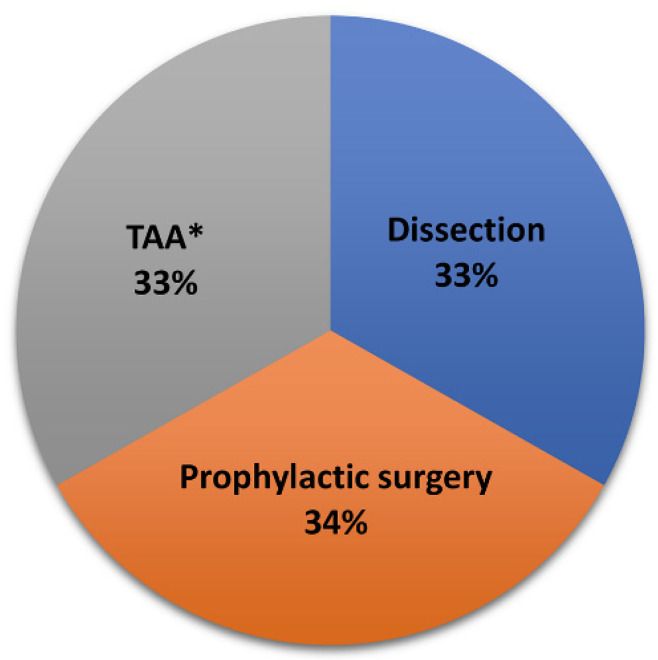
Grouping of cases (*N* = 241) into clinical endpoint categories. This study includes cases with history of thoracic aortic dissection (*N* = 80), prophylactic thoracic aortic repair surgery (*N* = 81), or thoracic aortic aneurysm (TAA) without prior dissection or surgery (*N* = 80). In the latter group, marked with an asterisk (*), the maximum proximal aortic diameter averaged 4.5 ± 0.4 cm.

A total of 241 participants with history of TAA or TAD involving the aortic root or ascending aorta met the inclusion criteria. Demographic and clinical characteristics are shown in Table 1. Eighty patients had a history of TAD (Figure 1), consisting of 61 with a type A dissection and 21 with a type B dissection; two patients had a type A and type B dissection occur asynchronously. All patients with history of type B dissection also had a history of TAA involving the root or ascending aorta. Among the 161 patients without prior TAD, 81 had a history of thoracic aortic repair surgery. Most of these were aortic root and/or ascending aorta repairs (*N* = 74). The seven patients who instead had history of prophylactic aortic arch or descending thoracic aorta repair also had TAA involving the root or ascending aorta. In total, 18 patients (7%) had a syndromic diagnosis that predisposes one to TAA. In addition, one patient had a diagnosis of Stickler syndrome, and one had adult onset Pompe disease. Pathogenic variants in *PRKG1* (p.R177Q) (*N* = 2) and *ACTA2* (p.R212G) (*N* = 1) were identified in three non-syndromic patients. The most common cardiovascular disease risk factors were hypertension (*N* = 186, 78%), dyslipidemia (*N* = 122, 51%), and obesity (body mass index > 30) (*N* = 99, 42%). History of smoking (*N* = 134, 56%), work or other regular activities that required heavy lifting (*N* = 48, 20%), and heavy weightlifting for the purpose of exercise (*N* = 23, 10%) were also reported.

### 3.2. Connective Tissue Abnormalities in Patients with TAA or TAD 

In total, 47% of patients reported at least one systemic connective tissue abnormality. These included abdominal hernia (20%), skin striae in atypical location (13%), hyperextensible skin (11%), hyperflexibility (10%), scoliosis (10%), atrophic scarring (8%), pectus carinatum (5%), and pectus excavatum (4%). Most patients with abdominal hernia required a surgical hernia repair (39 of 47), and three had recurrence of the hernia after surgery. The locations and frequencies of atypical striae are specified in Table 2. Scoliosis was diagnosed during childhood in 13 of the 24 cases. In the subgroup of patients who were not diagnosed with a TAA-associated syndrome (*N* = 223), 45% had at least one systemic connective tissue finding. These included abdominal hernia (20%), striae in atypical location (10%), hyperextensible skin (9%), atrophic scarring (8%), scoliosis (8%), hyperflexibility (7%), pectus carinatum (3%), and pectus excavatum (3%). Thus, non-cardiovascular connective tissue abnormalities were present in nearly one half of this adult TAA population, including in nearly half of those who did not have a diagnosis of a TAA-associated genetic syndrome.

### 3.3. Characteristics Associated with TAD 

**Figure 2:**
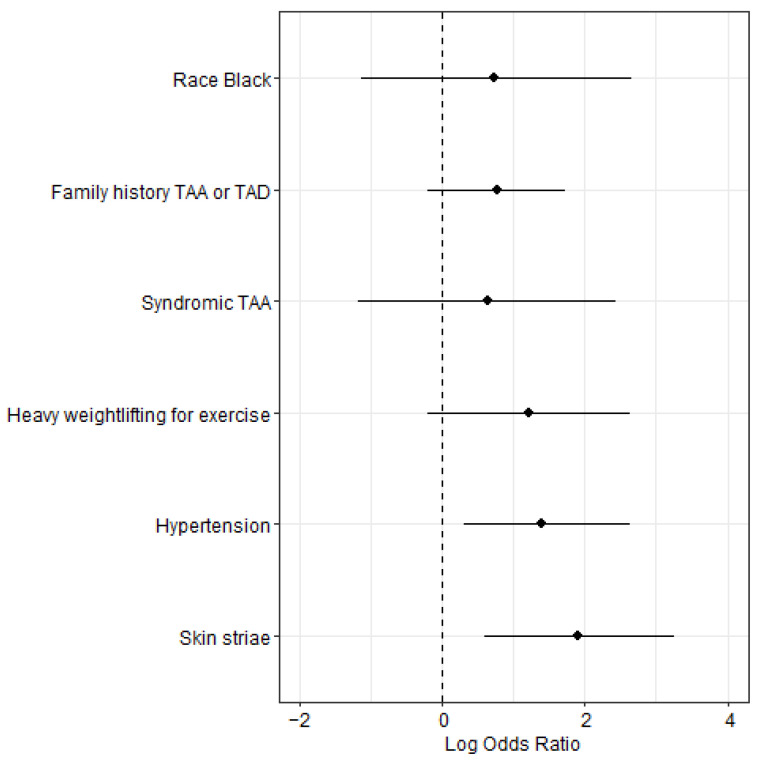
Result of multivariate analysis for characteristics associated with thoracic aortic dissection (TAD). Odds ratios are estimated through multivariate logistic regression model. Forest plot displays log_2_ of odd ratios (diamond) and 95% confidence intervals (bar). Skin striae (*p* = 0.005) and hypertension (*p* = 0.018) were independently associated with TAD. TAA: thoracic aortic aneurysm.

The characteristics that were significantly more frequent in TAD compared with non-TAD cases in univariate comparisons were Black or African American race, family history of TAA or TAD, genetic syndrome that predisposes one to TAA, hypertension; history of heavy weightlifting for exercise, and skin striae in atypical location (Table 3). Multivariate logistic regression analysis that included these six characteristics identified that striae (*p* = 0.005) and hypertension (*p* = 0.018) were independently associated with TAD (Figure 2). Inclusion of enrollment age and sex in the regression analysis did not change this result. A comparison between TAD cases and sex- and age-matched controls (32 matched participants per group from a pool of 84 eligible controls) also identified a significantly increased frequency of striae in TAD cases (*p* = 0.0044 by McNemar’s test). Meanwhile, there was not a significant increase in striae in TAA cases without TAD compared with controls. Overall, the findings suggest that striae and hypertension were risk factors for development of TAD in this study population.

### 3.4. Characteristics Associated with TAD in Patients without a Syndromic Diagnosis

Next, the 18 patients with an established diagnosis of MFS, LDS, or other TAA-associated syndrome were excluded. The remaining 223 cases included 70 TAD cases and 153 non-TAD cases. Their clinical and connective tissue characteristics are shown in Table S1. The prevalence of scoliosis, pectus excavatum, pectus carinatum, and mitral valve prolapse in these 223 cases were markedly lower than previously reported in a large cohort of adults with MFS [10] and cohorts with LDS [11,12]. Consistent with findings in the overall cohort, in this subset of patients, striae were more frequent in TAD cases (22%) versus non-TAD cases (5%), with an odds ratio (OR) of 5.7 and 95% confidence interval (CI) 2.2–14.7 (Table S1). In multivariate logistic regression, striae (*p* = 0.0004), family history of TAA or TAD (*p* = 0.010), and hypertension (*p* = 0.015) were independently associated with TAD (Figure S1). Meanwhile, the cumulative number of revised Ghent systemic points for MFS was not significantly associated with TAD (Table 4). Further analysis excluded all patients with systemic points for MFS or connective tissue characteristics (abdominal hernia, hyperextensible skin, hyperflexibility, wide atrophic scars) other than striae. In this subset, striae were present in 11 of 41 patients with TAD (27%) versus 4 of 90 patients without TAD (4%), indicating significantly increased OR of 7.9 with CI 2.3–26.6 (*p* = 0.0002). These results indicate that striae in an atypical location may be a marker of TAD risk in patients who do not have a syndromic diagnosis, whereas other systemic features of MFS may be less informative in the calculation of TAD risk.

### 3.5. Striae and Age at Clinical Endpoint

**Figure 3:**
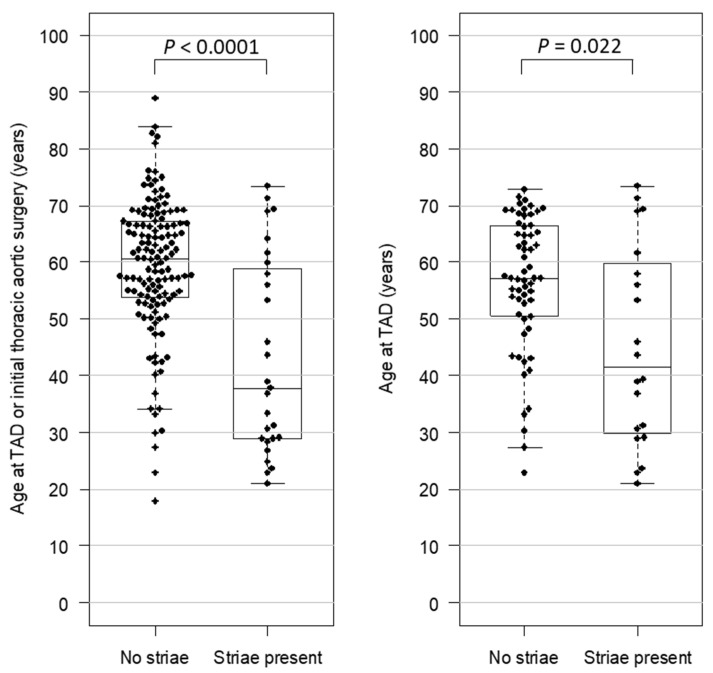
Comparison of age at clinical endpoints between cases with or without striae. Left graph displays ages at initial thoracic aortic dissection (TAD) or initial thoracic aortic repair. Right graph only displays ages at initial TAD. Each dot corresponds to one participant. Boxes represent the median and 1st and 3rd quartiles of ages for each group. Groups were compared using the Wilcoxon rank sum test.

Among the 161 cases who reached a clinical endpoint, defined as TAD or prophylactic aortic surgery, the initial events occurred at younger ages in patients with striae (mean 43 ± 17 years) than without striae (mean 59 ± 12 years) (*p* < 0.0001) (Figure 3). The result was similar after excluding the 16 patients with a syndromic diagnosis who reached one of these endpoints (*p* = 0.014). Among only the cases who had TAD, striae were associated with younger age at initial TAD (*p* = 0.022) (Figure 3). These findings indicate that in patients with aortopathy, striae are associated with earlier progression to clinical endpoints.

### 3.6. Systemic Features of MFS or LDS in Patients with Striae

The revised Ghent systemic criteria for MFS that were ascertainable in this study and the locations of striae were tabulated for each participant with striae (Table S2). In cases without a diagnosis of MFS or LDS, only 7 of 22 patients with striae received an additional systemic point. In comparison, eight of the nine patients with striae and a diagnosis of MFS or LDS had at least one additional point, and seven of these had two or more additional points. Club foot and cleft palate, which are associated with LDS [13], were identified in one patient each. Neither patient had arterial tortuosity, which is common in LDS, and the cleft palate was likely due to the diagnosis of Stickler syndrome [14]. These results support a low level of suspicion for undiagnosed MFS or LDS in striae cases who did not have clinical genetic testing.

## 4. Discussion

In this study, we characterized systemic connective tissue abnormalities in an adult population with trileaflet aortic valve and TAA or TAD involving the proximal aorta. The main findings are that (1) connective tissue abnormalities were prevalent and variable, (2) striae were specifically associated with TAD, and (3) aortic events, including TAD or prophylactic aortic surgery, occurred at younger ages in patients with striae.

### 4.1. Connective Tissue Findings Point to Common Systemic Derangement in TAA

Nearly one half of patients in this study reported one or more connective tissue findings. These included systemic features of MFS that are in the revised Ghent nosology, as well as other connective tissue findings such as hyperflexibility, skin hyperextensibility, and atrophic scarring. Overall, the data suggest that a significant proportion of adults presenting with TAA have an underlying systemic connective tissue derangement. This comprises not only patients with established genetic disorders such as MFS or LDS, but also those with connective tissue findings of unknown etiology. Cardiac screening of young individuals who present with one or more of these features may help to identify asymptomatic TAA early. Other clinical characteristics that may help to identify patients with asymptomatic TAA include renal cysts and intracranial aneurysm [15], as well as the isolated connective tissue finding of a thumb-palm sign [16]. The development of new strategies to identify silent TAA in an effective manner is an important area of investigation.

### 4.2. Association between Striae and TAA Severity

We previously described prevalent systemic connective tissue findings in a cohort of pediatric patients with TAA who had non-diagnostic genetics evaluations [4]. Approximately 20% of those patients had striae, which were associated with increased rate of aortic root dilation. Striae were also previously identified as a possible risk factor for dissection in a small cohort of patients with MFS [17]. The results of the current study strengthen the evidence that striae may be associated with increased risk for progression to TAD in patients with TAA. The genetic or epigenetic factors that control the inter-individual variability of TAA severity are not well defined but likely myriad. Extracellular matrix dysregulation is characteristic of both striae [18,19,20] and aortic tissues with TAA [21]. Intracellular biomechanical dysfunction is observed in aortic smooth muscle cells in TAA and in fibroblasts derived from striae [22,23]. We postulate that in some patients, striae may be an overt phenotypic representation of the complex interplay between genetic and epigenetic processes that are occurring within the aorta.

### 4.3. Clinical Rationale for Utilizing Striae as a Predictor of TAA Progression to TAD

Early diagnosis of TAA facilitates monitoring, medical therapy, and surgical intervention before the development of a TAD. Cardiac imaging with echocardiography of young patients who present with striae in atypical locations may facilitate early diagnosis. Because TAA can develop silently in adulthood, the finding of striae may justify follow-up imaging at older ages when initial cardiac imaging is normal. Current aortic size criteria for prophylactic aortic surgery are imperfect predictors of TAD risk at the individual level [24], and there is a major need to develop new approaches to classify risk. Incorporating striae into risk classification algorithms may be an avenue toward improving medical and surgical decision making. Systematic tools to identify connective tissue findings in TAA patients, such as administration of standardized questionnaires routinely during cardiac care encounters, may better detect patients at higher risk and also prioritize referrals for complete genetics evaluations. In patients presenting acutely, striae in atypical locations could increase suspicion for a TAD. In clinical scenarios where genetic testing is unfeasible, striae could be prioritized above other systemic connective tissue findings to stratify risk. The interview-based approach undertaken in this study is a feasible alternative in clinical settings where challenges such as cost, genetics provider availability, and patient preferences restrict the ability to carry out a genetic evaluation. The data presented in this study are compelling, but further study in larger cohorts is necessary before widespread clinical application of the findings.

### 4.4. Hypertension, Family History and TAD

In this study, hypertension was independently associated with TAD, confirming prior evidence that it is a risk factor for TAD and supporting the use of antihypertensives in TAA [25]. This study also replicates prior evidence that approximately 20% of TAA is familial [26]. We observed that TAD was more frequent in familial TAA, which aligns with guidelines that recommend lower surgical thresholds in familial TAA [3]. Mutations in smooth muscle cell contractile genes, such as *ACTA2*, *MYH11*, *PRKG1*, and *MYLK*, cause familial TAA in the absence of connective tissue findings [22]. The data are consistent with knowledge that multiple pathways have a role in TAA pathogenesis, which have variable degrees of non-aortic involvement, and underscore the importance of cardiac screening of first-degree relatives of all patients with TAA to identify asymptomatic disease early. 

A limitation of this study is that participants were enrolled from a single center. Enrollment occurred in the setting of cardiothoracic surgical care. Therefore, the cohort was enriched with relatively severe cases, but this likely represents the patient populations routinely evaluated in other surgical practices. Studying this patient pool facilitated the analysis of clinical endpoints. Most study participants were not examined by a geneticist for connective tissue features. Therefore, some of the revised Ghent systemic criteria for MFS, such as facial dysmorphism or hindfoot deformity, were not ascertained. Dermatological examinations were not directly performed in this study. The frequency of striae in our study’s controls matches a prior study that included dermatological examination [7]. This suggests that study interviews were able to identify striae reliably. In our secondary analysis, only a subset of cases and controls were able to be age- and sex-matched. This may have limited the ability to detect differences between controls and the TAA cases who did not have TAD. Future studies that include prospective physical examination of striae in cases and matched controls in greater numbers are warranted. Deep phenotyping of striae quality, location, and extent may help to discriminate striae characteristics that most strongly represent an intrinsic connective tissue derangement. This study excluded patients with bicuspid aortic valve-related aortopathy because auscultatory findings may lead to earlier diagnosis than in patients with clinically silent aortopathy, and aortic repairs may be performed earlier when there is a primary indication of aortic valve surgery. Future investigation of connective tissue findings in the context of bicuspid aortic valve may be informative. 

The findings in this study deserve careful interpretation because the connective tissue data were collected using interviews and not by direct physical examination. One strength of the approach is feasibility for research and clinical implementation, but subjectivity in participant responses is a potential weakness. The study’s sample size was relatively small but enriched for relatively severe cases. Replication in a larger cohort that has full clinical evaluation by a geneticist is necessary to conclusively determine the association between striae and TAD, and whether this association is independent of the presence of an undiagnosed genetic syndrome. 

## 5. Conclusions

In TAA, the connective tissue finding of skin striae may be a clinical indicator for increased risk for early progression and dissection. Risk stratification is important to optimize clinical decisions on medical therapy, activity restrictions, frequency of follow up, and prophylactic surgery. Prospective studies that include detailed connective tissue phenotyping and molecular testing are warranted in order to develop risk classification models that provide more individualized clinical care. Identification of shared molecular and cellular mechanisms between striae and aortopathy may help to elucidate TAA pathogenesis.

## Figures and Tables

**Table 1:** Demographic and clinical characteristics in study cases (*N* = 241).

Characteristic	Value
Enrollment age (year), mean ± SD	61 ± 13
Sex, *n* (%)	
Male	175 (73)
Female	66 (27)
Race, *n* (%)	
White	226 (94)
Black or African American	13 (5)
Asian	2 (0.8)
Ethnicity, *n* (%)	
Non-Hispanic	239 (99)
Hispanic	2 (1)
Genetic syndrome associated with TAA/TAD, *n* (%)	18 (7)
Marfan, *n*	14
Loeys-Dietz, *n*	2
Vascular Ehlers-Danlos, *n*	1
Turner, *n*	1
Family history of TAA/TAD, excluding syndrome cases, *n* (%)	

SD: standard deviation; TAA: thoracic aortic aneurysm; TAD: thoracic aortic dissection.

**Table 2:** Locations and frequency of skin striae classified as atypical (*N* = 31 cases).

Location	Number of Cases (% of Total Cases)
Abdomen *	14 (6)
Shoulder	9 (4)
Arm	6 (2)
Back	6 (2)
Chest *	5 (2)
Axilla	2 (1)
Flank	1 (0.4)
Diffuse	3 (1)

* Women with history of pregnancy were excluded from the count.

**Table 3:** Comparison of clinical and connective tissue characteristics between cases with thoracic aortic dissection (TAD) (*N* = 80) versus cases without TAD (*N* = 161).

Characteristic	TAD, *N* (%)	No TAD, *N* (%)	OR (95% CI)	*p*-Value
Sex male	58 (73)	117 (73)	1 (0.5–1.8)	0.98
**Race Black or African American**	**8 (10)**	**5 (3)**	**3.5 (1.1–11.0)**	**0.026**
**Family history of TAA or TAD**	**28 (35)**	**33 (21)**	**2.1 (1.1–3.8)**	**0.015**
**Syndromic diagnosis**	**10 (13)**	**8 (5)**	**2.7 (1.0–7.2)**	**0.036**
**Hypertension**	**68 (86)**	**118 (74)**	**2.2 (1.1–4.6)**	**0.031**
Dyslipidemia	37 (47)	85 (53)	0.8 (0.5–1.4)	0.44
Obesity, BMI > 30	33 (42)	66 (41)	1.0 (0.6–1.8)	0.88
Type 2 diabetes mellitus	10 (13)	20 (13)	1.0 (0.5–2.3)	1.0
Coronary artery disease	22 (28)	46 (29)	0.9 (0.5–1.7)	0.86
Stroke	12 (15)	15 (9)	1.7 (0.8–3.9)	0.19
History of cigarette smoking	48 (60)	86 (54)	1.3 (0.7–2.2)	0.36
**Heavy weightlifting for exercise**	**13 (17)**	**10 (6)**	**3.0 (1.2–7.1)**	**0.011**
Heavy lifting for other activities	18 (23)	30 (19)	1.3 (0.7–2.5)	0.45
Mitral valve prolapse	4 (5)	9 (6)	0.9 (0.3–3.0)	0.86
Abdominal hernia	12 (15)	35 (22)	0.6 (0.3–1.3)	0.21
Inguinal	5 (6)	24 (15)	NA	NA
Umbilical	7 (9)	12 (7)	NA	NA
Femoral	1 (1)	2 (1)	NA	NA
**Skin striae**	**20 (25)**	**11 (7)**	**4.5 (2.0–10.0)**	**<0.0001**
Hyperextensible skin	8 (10)	17 (11)	1.0 (0.4–2.3)	0.93
Hyperflexibility	11 (14)	13 (8)	1.8 (0.8–4.3)	0.17
Scoliosis	7 (9)	17 (11)	0.8 (0.3–2.0)	0.66
Wide atrophic scars	10 (13)	9 (6)	2.4 (0.9–6.2)	0.063
Pectus carinatum	6 (8)	6 (4)	2.2 (0.7–7.0)	0.18
Pectus excavatum	3 (4)	7 (4)	0.9 (0.2–3.5)	0.86

Groups were compared using chi-squared tests. Characteristics with *p* value < 0.05 are highlighted in bold. BMI: body mass index; CI: confidence interval; NA: Not assessed; OR: odds ratio.

**Table 4:** Comparison of total revised Ghent systemic points between thoracic aortic dissection (TAD) (*N* = 70) and non-TAD (*N* = 153) cases who lacked a diagnosis of a TAA-associated syndrome.

	Striae Included in Calculation of Points			Striae Not Included in Calculation of Points		
Number of Revised Ghent Systemic Points	TAD, *n* (%)	No TAD, *n* (%)	*p* Value	TAD, *n* (%)	No TAD, *n* (%)	*p* Value
0	47 (67)	127 (83)	0.16	58 (83)	131 (86)	0.89
1	17 (24)	15 (10)		8 (11)	12 (8)	
2	4 (6)	7 (5)		4 (6)	6 (4)	
3	2 (3)	2 (1)		0	3 (2)	
4	0	1 (0.7)		0	1 (0.7)	
5	0	1 (0.7)		0	0	

Cases with Marfan syndrome, Loeys-Dietz syndrome, vascular Ehlers-Danlos syndrome, or Turner syndrome are not included. The highest ascertainable number of points in this study was 7. TAD and non-TAD groups were compared using the Cochran Armitage Trend Test.

## Data Availability

The study did not utilize public archive datasets. Data inquiries should be sent to the corresponding author, B.J.L.

## Supplementary Materials

The following are available online at https://www.mdpi.com/article/10.3390/genes13010023/s1, Figure S1: Result of multivariate analysis for characteristics associated with thoracic aortic dissection (TAD) among 223 cases who did not have a clinical diagnosis of Marfan, Loeys-Dietz, vascular Ehlers-Danlos, or Turner syndromes. Table S1: Frequency of characteristics between TAD (*N* = 70) or non-TAD cases (*N* = 153) who did not have an established diagnosis of Marfan, Loeys-Dietz, vascular Ehlers-Danlos, or Turner syndrome. Table S2: Characteristics of study patients with striae in atypical location (*N* = 31), stratified by history of thoracic aortic dissection (TAD) status.
