# Supplementary material for: An Emergent Nexus between Striae and Thoracic Aortic Dissection
Source: Genes (Basel). 2021 Dec 23;13(1):23. doi: 10.3390/genes13010023 (PMC8774627; doi:10.3390/genes13010023)
Supplement: Supplementary file 1 [file genes-13-00023-s001.zip › Figure S1.pdf]

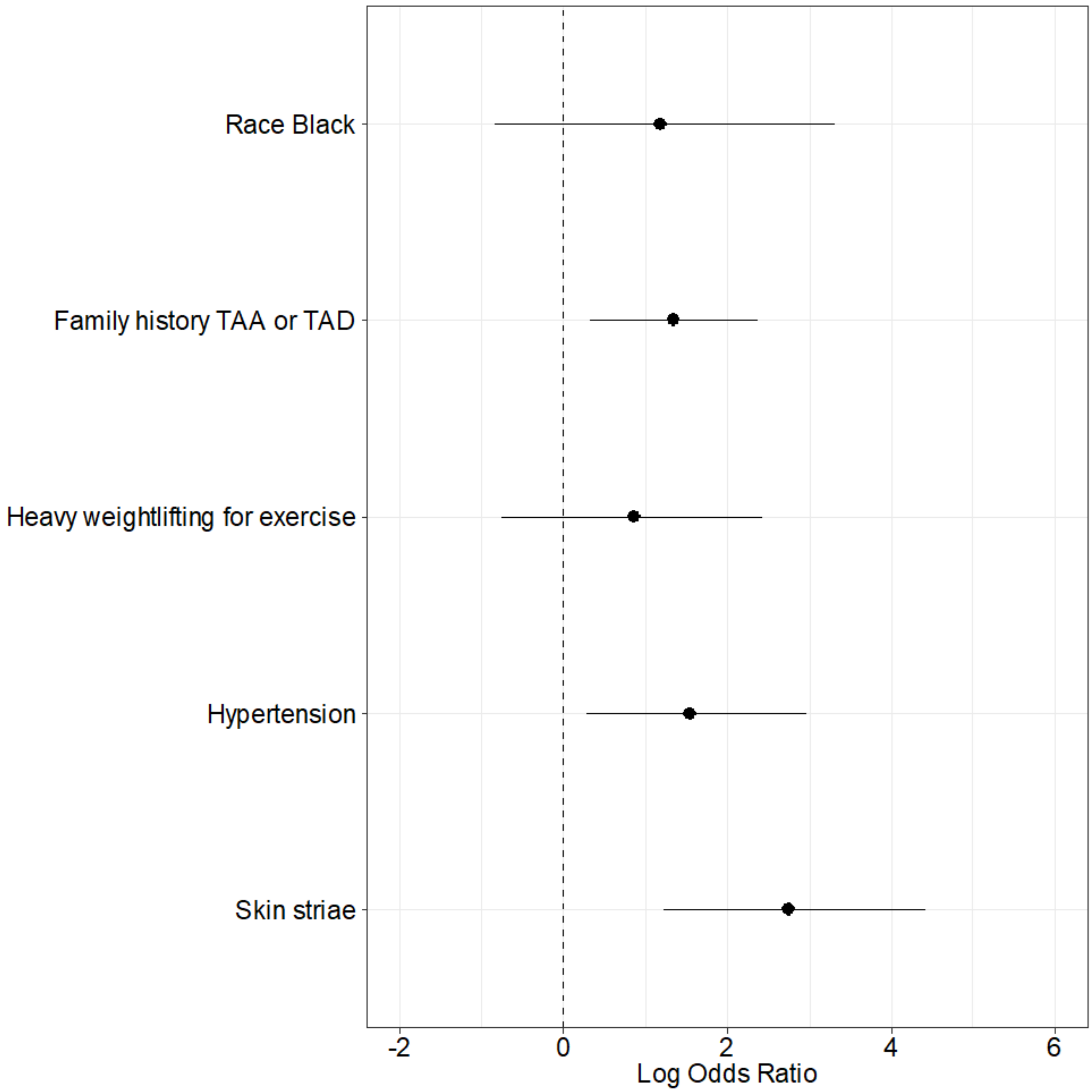

Figure S1. Result of multivariate analysis for characteristics associated with thoracic aortic dissection (TAD) among 223 cases who did not have a clinical diagnosis of Marfan, Loeys-Dietz, vascular Ehlers-Danlos, or Turner syndromes. Odds ratios are estimated through multivariate logistic regression model. Forest plot displays log2 of odd ratios (diamond) and 95% confidence intervals (bar). Skin striae (p=0.0004), family history of thoracic aortic aneurysm (TAA) or TAD (p=0.010), and hypertension (p=0.015) were independently associated with TAD.
