# Supplementary material for: An Emergent Nexus between Striae and Thoracic Aortic Dissection
Source: Genes (Basel). 2021 Dec 23;13(1):23. doi: 10.3390/genes13010023 (PMC8774627; doi:10.3390/genes13010023)
Supplement: Supplementary file 1 [file genes-13-00023-s001.zip › Table S1.pdf]

Table S1. Frequency of characteristics between TAD (N=70) or non-TAD cases (N=153) who did not have an established diagnosis of Marfan, Loeys-Dietz, vascular Ehlers-Danlos, or Turner syndrome.

Characteristic	TAD, N (%)	No TAD, N (%)	OR [95% CI]	P value
Sex male	51 (73)	112 (73)	1.0 [0.5-1.9]	0.96
**Race Black or African American**	**8 (11)**	**4 (3)**	**4.8 [1.4-16.5]**	**0.0068**
**Family history of TAA or TAD**	**24 (34)**	**25 (16)**	**2.7 [1.4-5.1]**	**0.0027**
**Hypertension**	**62 (89)**	**115 (76)**	**2.5 [1.1-5.7]**	**0.026**
Hyperlipidemia	34 (50)	83 (54)	0.8 [0.5-1.5]	0.56
Obesity (BMI > 30)	29 (43)	65 (43)	1.0 [0.6-1.8]	0.99
Type 2 diabetes mellitus	9 (13)	19 (12)	1.0 [0.4-2.4]	0.93
Coronary artery disease	21 (30)	46 (30)	1.0 [0.5-1.8]	0.99
Stroke	10 (14)	14 (9)	1.7 [0.7-3.9]	0.25
History of cigarette smoking	45 (64)	83 (55)	1.5 [0.8-2.7]	0.18
Heavy weightlifting for exercise	10 (15)	10 (7)	2.4 [1.0-6.2]	0.055
Heavy lifting for other activities	15 (22)	27 (18)	1.3 [0.6-2.6]	0.47
Mitral valve prolapse	3 (4)	6 (4)	1.1 [0.3-4.5]	0.91
Abdominal hernia	11 (16)	33 (22)	0.7 [0.3-1.4]	0.31
Inguinal	4 (6)	24 (16)		
Umbilical	7 (10)	11 (7)		
Femoral	1 (1)	1 (1)		
**Skin striae**	**15 (22)**	**7 (5)**	**5.7 [2.2-14.7]**	**<0.0001**
Hyperextensible skin	6 (9)	14 (9)	1.0 [0.4-2.6]	0.90
Hyperflexibility	6 (9)	9 (6)	1.5 [0.5-4.4]	0.46

Scoliosis	5 (7)	13 (9)	0.8 [0.3-2.4]	0.73
Wide atrophic scars	9 (13)	9 (6)	2.4 [0.9-6.2]	0.078
Pectus excavatum	2 (3)	4 (3)	1.1 [0.2-6.3]	0.89
Pectus carinatum	2 (3)	5 (3)	0.9 [0.2-4.8]	0.91

Characteristics with p value < 0.05 are highlighted in bold.

BMI: Body mass index; CI: Confidence interval; OR: odds ratio.
