# Supplementary material for: An Emergent Nexus between Striae and Thoracic Aortic Dissection
Source: Genes (Basel). 2021 Dec 23;13(1):23. doi: 10.3390/genes13010023 (PMC8774627; doi:10.3390/genes13010023)
Supplement: Supplementary file 1 [file genes-13-00023-s001.zip › Table S2.pdf]

Table S2. Characteristics of study patients with striae in atypical location (N=31), stratified by history of thoracic aortic dissection (TAD) status.

| ID | TAD | Sex | Age | Race | Genetic eval or testing | Syndrome | Fam Hx TAA or TAD | MVP | Pectus Car/Exc | Scoliosis | PTX | Striae | Systemic point total* | Shoulder striae | Arm striae | Axilla striae | Chest striae | Back striae | Abdomen striae | Flank striae | Diffuse striae |
|---|---|---|---|---|---|---|---|---|---|---|---|---|---|---|---|---|---|---|---|---|---|
| 0210 | No | F | 38 | B | Yes | MFS | + | + | -/+ | + | | + | 4 | + | + | | | | | | |
| 0436 | No | F | 63 | W | Yes | MFS | + | + | | + | | + | 3 | | | | | | | | + |
| 0714 | No | M | 43 | W | Yes | MFS | + | | | + | | + | 2 | | + | | | + | + | | |
| 0131 | No | M | 34 | W | Yes | LDS | + | | +/- | + | | + | 4 | + | | | | | + | | |
| 0813 | No | F | 39 | W | Yes | Stickler | | | +/+ | | | + | 4 | | + | | + | + | | | |
| 0064 | No | M | 60 | W | No | None | | | | | | + | 1 | | | | | | + | | |
| 0226 | No | M | 35 | W | No | None | | + | +/- | + | | + | 5 | | | | | | + | | |
| 0290 | No | M | 65 | W | No | None | | | -/+ | | | + | 2 | | | | | | | + | |
| 0311 | No | M | 48 | B | No | None | | | | | | + | 1 | | + | | | | + | | |
| 0333 | No | M | 38 | W | No | None | | | | | | + | 1 | | | | | | + | | |
| 0415 | No | M | 53 | W | No | None | | | | | | + | 1 | | | + | | | + | | |
| 0012 | Yes | M | 38 | W | Yes | MFS | | | +/- | + | | + | 4 | | | | | + | | | |
| 0198 | Yes | M | 31 | W | Yes | MFS | + | | +/- | | | + | 3 | + | | | | | | | |
| 0252 | Yes | M | 38 | W | Yes | MFS | | | | | | + | 1 | + | | | | + | | | |
| 0264 | Yes | M | 41 | W | Yes | MFS | | NA | +/- | + | | + | 4 | + | | | + | | | | |
| 0301 | Yes | M | 33 | W | Yes | MFS | + | + | -/+ | | | + | 3 | + | | | + | | + | | |
| 0086 | Yes | M | 67 | W | No | None | + | | | | | + | 1 | + | | | | | + | | |
| 0105 | Yes | F | 55 | B | Yes | None | + | | | | | + | 1 | | | | + | | | | |
| 0114 | Yes | M | 83 | W | No | None | | | | | | + | 1 | | + | | | | | | |
| 0122 | Yes | M | 46 | W | No | None | + | | | | | + | 1 | | | + | | | + | | |
| 0141 | Yes | M | 33 | W | No | None*** | + | | | + | | + | 2 | | | | | | + | | |
| 0220 | Yes | M | 70 | W | No | None | | | | | + | + | 3 | | | | | | + | | |
| 0245 | Yes | M | 75 | W | No | None | | | | | | + | 1 | | | | | | | | + |
| 0337 | Yes | M | 39 | B | NA | None | | | +/- | | | + | 3 | + | | | | + | | | |
| 0369 | Yes | M | 24 | W | Yes | None** | | | | | | + | 1 | | | | | + | | | |
| 0445 | Yes | M | 72 | W | No | None | | | | | | + | 1 | | | | | | + | | |
| 0475 | Yes | M | 59 | W | No | None | + | | | | | + | 1 | | + | | | | | | |
| 0480 | Yes | F | 58 | B | No | None | | | | | | + | 1 | | | | | | | | + |
| 0552 | Yes | M | 71 | W | No | None | | | | | | + | 1 | | | | | | + | | |
| 0573 | Yes | M | 48 | B | Yes | None | | | -/+ | | | + | 2 | + | | | | | | | |
| 0753 | Yes | M | 31 | W | Yes | None | | | | | | + | 1 | | | | + | | | | |

Cases without history of TAD are shaded green and cases with history of TAD are shaded pink.

*Striae is included in the calculation of revised Ghent systemic points; **Carries likely pathogenic variant in *ACTA2*; ***May carry a pathogenic variant in *ACTA2* that has been identified in multiple affected relatives.

B=Black or African American; Car=Carinatum; Exc=Excavatum; F=female; Hx=history; LDS=Loeys-Dietz syndrome; M=male; MFS=Marfan syndrome; NA=data not available; PTX=spontaneous pneumothorax; TAA=thoracic aortic aneurysm; W=White
